# One-stage anterior approach for long-segment subaxial cervical spondylitis tuberculosis: A case report

**DOI:** 10.1016/j.ijscr.2022.107693

**Published:** 2022-09-21

**Authors:** Rifqi Aulia Destiansyah, Eko Agus Subagio, Abdul Hafid Bajamal, Muhammad Faris

**Affiliations:** Department of Neurosurgery, Universitas Airlangga – Dr. Soetomo General Academic Hospital, Surabaya, East Java, Indonesia

**Keywords:** Cervical spondylitis, Tuberculosis, Surgery, Anterior approach, Extrapulmonary TB

## Abstract

**Background:**

Spondylitis TB on cervical region is a rare disease, that may lead to severe neurological complications. The anterior approach is considered as a gold standard for cervical spine tuberculosis. Available studies and literature have not precisely mentioned on how many levels are acceptable for this disease and still up for discussion.

**Case presentation:**

A 45-year-old Asian male was brought from a rural hospital to our outpatient clinic with progressive weakness of all extremities for 3 months. Cervical x-ray and MRI showed three-levels of vertebral body destruction, suggesting a cervical spondylitis TB. Patient had debridement, corpectomy on C4, 5, 6, fusion with cage, and anterior plating from C3 to Th1 in a one-stage anterior approach. Immediately after the surgery, the patient had no complaints of pain, and he was able to walk on his own. One year follow-up after the surgery, no residual neurological impairment is detected and had no limitation in daily activities. Cervical x-ray and MRI showed good ossification and improvement of lordotic curvature.

**Conclusion:**

Treatment of cervical spondylitis TB which involved three-levels of vertebrae using one-stage anterior approach provides a good rate of deformity correction along with clinical improvement and long-term well-being of the patient.

## Highlights

• Cervical TB is rare and could lead to severe neurological symptoms, thus, should be diagnosed and managed with extra care.
• The anterior approach is considered as a gold standard for cervical spine tuberculosis, since the disease usually involves anterior column, allowing direct access to the focus of the disease, and provide better stabilization
• Opinions in the literature regarding the management of cervical spondylitis TB have periodically fluctuated from completely conservative to radical surgical management.
• In this case the use of anterior approach for involvement of 3 levels of cervical vertebrae is to correct the kyphosis of the patient.
• Treatment of cervical spondylitis TB which involved three-levels of vertebrae using one-stage anterior approach provides a good rate of deformity correction along with clinical improvement of the patient.

## 1 Introduction

Tuberculosis (TB) remained a significant disease burden and causes huge economic loss worldwide. Indonesia has a numerous TB prevalence, with 759 out of 100,000 people (95 % CI: 589,7-960,8) suffering from this disease. 60 % of the new TB cases worldwide, are accounted from 6 countries, including Indonesia [1]. Spinal tuberculosis is one form of extrapulmonary TB frequently observed. At present, there is no data about the incidence and prevalence of spinal tuberculosis, both worldwide and per country [2].

Spinal tuberculosis (Pott's disease) on the cervical region is a rare incidence, varying from 2 to 12 % of all cases of spinal tuberculosis. Cervical TB could lead to severe neurological symptoms, thus, should be diagnosed and managed with extra care. Surgical approaches to cervical spinal tuberculosis are still contentious. There are 3 surgical approaches available: anterior, posterior, and both (360^0^). The anterior approach is considered a gold standard for cervical spine tuberculosis, since the disease usually involves the anterior column, allowing direct access to the focus of the disease, and providing better stabilization [3]. However, available studies and literature nowadays have not precisely mentioned how many levels are acceptable for this approach, thus, procedures involving more than two levels of vertebrae are still up for debate. We present a case with a 45-years old male with cervical spine tuberculosis managed with merely one stage of anterior approach with a good outcome, with a one-year follow-up without any pulmonary TB symptoms. This article is reported based on SCARE 2020 [10].

## 2 Case presentation

A 45-years-old Asian male patient came to our academic general hospital outpatient clinic, referred from a countryside hospital. The patient suffered from weakness on all extremities for 3 months before admission. Patient realized the symptoms got worse when driving a car and he was unable to walk for 1 month. Two weeks later, patient felt weakness on upper extremities, alongside a tingling sensation. Patient also complained of neck pain. Pain was radiating to both hands and legs. There was no history of fever, decreased body weight, long period of cough and contact with TB patients. However, there was night sweating for 5 months before admission. History of trauma was denied. There were no disturbances on urination and defecation.

From physical examination, we found normal vital sign. Neurological examination showed tetra-paresis with motoric power of 3 on all extremities, hypoesthesia on C4 level and below, increased physiologic reflexes and positive pathologic reflexes (Babinski and Chaddock) on both sides with normal anal sphincter tones and bulbocavernosus reflex.

**Fig. 1:**
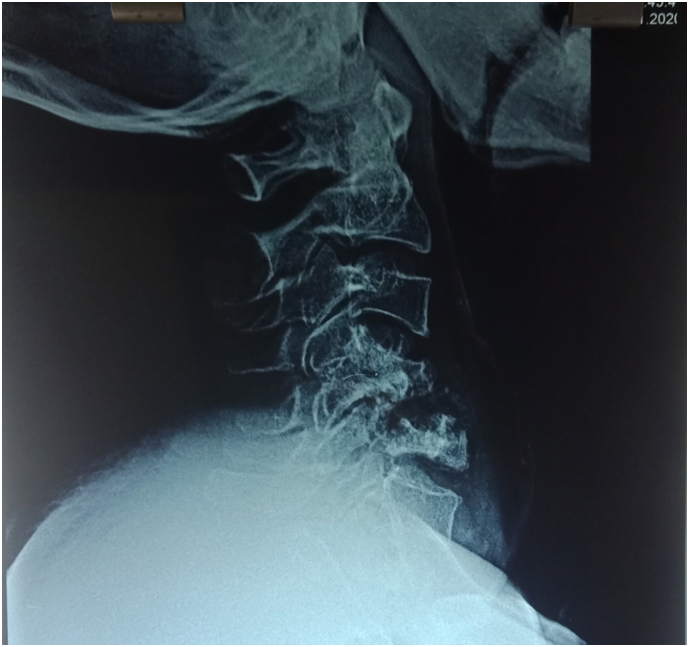
Pre-operative lateral cervical X-ray.

**Fig. 2:**
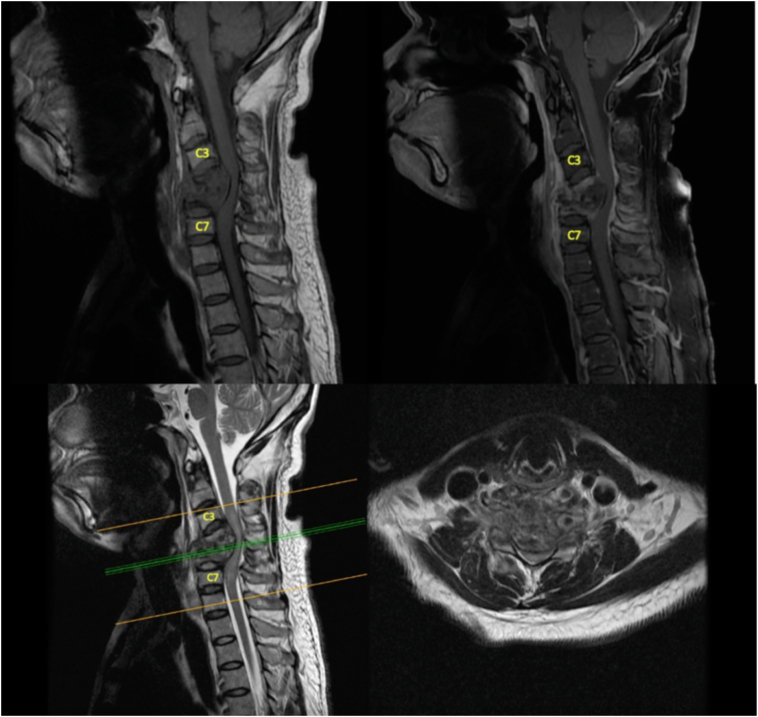
Cervical MRI, T1 with contrast and T2 Sequence.

We did several radiologic examinations for attaining a diagnosis. Cervical x-ray showed destruction on C4, 5 and 6, kyphotic curvature with Cobb's angle of 8.5° (Fig. 1). Cervical MRI with contrast showed three-level vertebral bodies and disc destructions at C4, C5 and C6 with hypointense mass on T1 and isointense on T2 at anterior C3, 4, 5, 6 and Th1, suspected as a cervical tuberculosis infection (Fig. 2), and regiment of TB drugs was subsequently given to the patient.

**Fig. 3:**
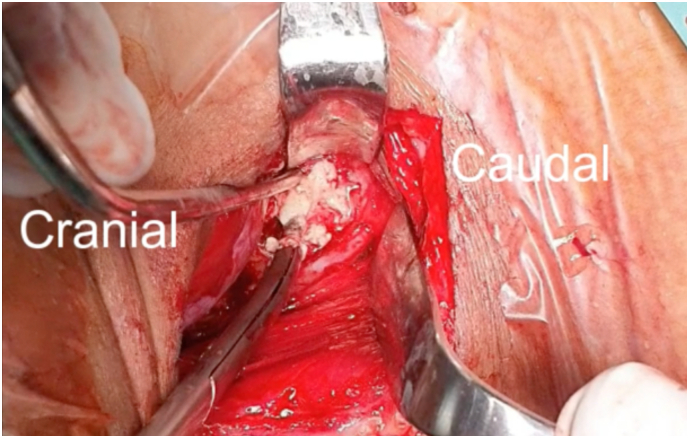
Anterior abscess showed up after dissection.

As the cord compression is located anteriorly and the aim was to correct kyphotic of the cervical curvature, we decided to do debridement, corpectomy on three-levels of C4, 5, and 6, fusion with cage and anterior plating from C3 to Th1, all with a one-stage anterior approach. After dissection was done, we found bony destructions on C4, 5, and 6, with the abscess flowing (Fig. 3). Abscess was evacuated and specimen was sent to microbiology for further examination (gram and specific staining for TB, culture), showed MTB with no Rifampicin resistance, thus, medication for extra pulmonary TB was continued until 9 months.

**Fig. 4:**
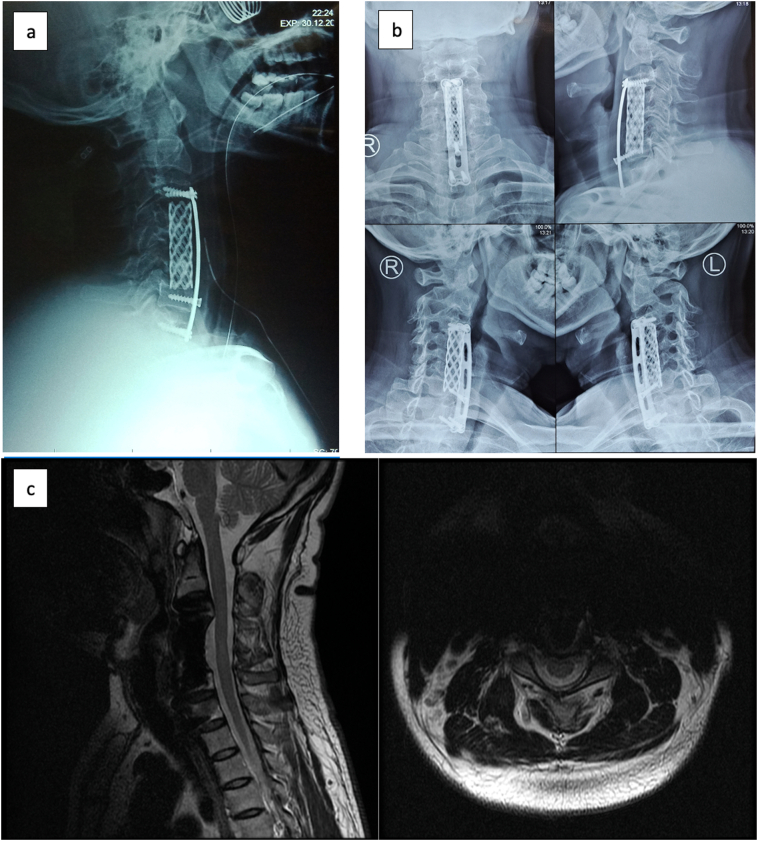
(a) Cervical X-Ray early post-operative; (b) 1-year post-operative follow-up Cervical X-ray; (c) 1-year post-operative follow-up Cervical MRI.

Corpectomy on three-levels on C4, 5, and 6 was done. Furthermore, instrumentation for anterior column reconstruction with cylindrical cage, bone grafting, and cervical plate was applied. A post-operative cervical x-ray showed the return of cervical lordosis curvature, placement of anterior instrumentation with hollow cage, screw and plating (Fig. 4a).

Immediately after the surgery, the patient had no complaints of pain, and he was able to walk on his own. One year follow-up after the surgery, no residual neurological impairment is detected, patient is able to run and had no limitation in daily activities. One year following the surgery the patient still had no complaints and able to conduct rigorous physical activity. Cervical x-ray and MRI were done and showed good ossification and improvement of lordotic curvature (Fig. 4b, c).

## 3 Discussion

Spinal TB (Pott's disease) is a detrimental form of TB and is common in young-adult, particularly in developing country like Indonesia. The World Health Organization (WHO) data, implied an envisioned incidence of 10 million new TB cases in 2018, of which Southeast Asia subsidized to 44 % of the worldwide cases [1]. Nevertheless, it is frequently missed or misdiagnosed because of inadequate standard health care centers, mainly in the remote areas [2]. Even though the occurrence of tuberculosis in general and spinal TB has less numbers in developed countries, it is far on the upwards thrust with most of these patients being immigrants from TB endemic regions [4]. In the UK, in over 6-years period, from 1999 to 2004, 729 patients had TB; of those, 8 % (61 cases) had musculoskeletal involvement, with 30 patients had spinal TB. Immigrants from endemic areas, such as the Indian subcontinent fashioned most of those patients [5].

Spine is involved in up to 50 % of patients with osteoarticular TB. Cervical spine is a complex anatomic region so that the structure is susceptible to any anomalies lies within [6]. Cervical spondylitis TB affected 4.2 to 12 % of all spinal TB cases. The resultant lesions lead to the development of cervical spine instability (CSI), neurological deficits, severe and progressive kyphosis, paraspinal and epidural abscess, and granuloma leading to cervicomedullary compression [7]. Cervical spondylitis TB grade was reported by Wang et al. on Xiangya Institutes of Medical sciences cervical tuberculosis grading system. In this study, it was described that grade 1 patients were initially managed conservatively, whereas grade 2 patients immediately underwent surgery A (anterior debridement/ decompression and fusion with autogenous bone grafting; Fig. 1), and grade 3 patients underwent PA/AP surgery (posterior fixation with instrumentation, followed by simultaneous or sequential anterior debridement/decompression and fusion [8].

Opinions in the literature regarding the management of cervical spondylitis TB have periodically fluctuated from completely conservative to radical surgical management. This transformation rose largely because many studies have been an isolated case report or because cervical spondylitis TB cases have been included in larger studies of tubercular afflictions of the skeletal system. Studies available to address specifically on cervical spondylitis TB generally reflected on surgeon's individual preference rather than based on clear guidelines. There are reports for anterior instrumentation and was accepted by spine surgeons worldwide, as in the cervical spondylitis TB, anterior part of the vertebral body; which is the most common focus of infection, thus, anterior decompression and instrumentation should be the reasonable procedure of choice [3].

However, the recent publications regarding anterior approach for cervical spondylitis TB has not been standardized on how many levels are acceptable for this procedure. Srivastava et al. published their study about anterior approach for cervical spondylitis TB of 46 patients, with the C5-C6 involvement of more than 50 % of the cases (24 of 46 cases) but none involve more than two levels of the vertebrae [9]. In our case, we decided to use the anterior approach with 3 levels of vertebrae, as the surgical indication was to correct the kyphosis of the patient. Pre-operative Cobb's angle was 8.5° kyphosis, which after the procedure became 13.5° lordosis. On the clinical significancy, there was an immediate neurological improvement on the patient.

## 4 Conclusion

Based on the result and outcome of this case, treatment of cervical spondylitis TB which involved three-levels of vertebrae using one-stage anterior approach including anterior debridement, corpectomy, instrumentation, and fusion, it can be concluded that this procedure provides a good rate of deformity correction along with clinical improvement and long-term well-being of the patient.

## Submission statement

This manuscript is original and has not been submitted elsewhere in part or in whole.

## Provenance and peer review

Not commissioned, externally peer-reviewed.

## Funding

None.

## Ethical approval

None.

## Consent

Written informed consent was obtained from the patient for publication of this case report and accompanying images. A copy of the written consent is available for review by the Editor-in-Chief of this journal on request.

## Author contribution

The roles are distributed as follows:

1. Conception and Design were done by RAD, MF, EAS AHB
2. Supervision was done by EAS AHB
3. Materials and data collection were done by RAD, MF
4. Analysis were done by RAD, MF
5. Literature Review and Editing were done by MF, EAS

## Registration of research studies

Not applicable

## Guarantor

MF accepts full responsibility for this review manuscript.

## Declaration of competing interest

None.
